# Inhibition of β-catenin signalling in dermal fibroblasts enhances hair follicle regeneration during wound healing

**DOI:** 10.1242/dev.131797

**Published:** 2016-07-15

**Authors:** Emanuel Rognoni, Celine Gomez, Angela Oliveira Pisco, Emma L. Rawlins, Ben D. Simons, Fiona M. Watt, Ryan R. Driskell

**Affiliations:** 1 King's College London Centre for Stem Cells and Regenerative Medicine, 28th Floor, Tower Wing, Guy's Hospital Campus, Great Maze Pond, London SE1 9RT, UK; 2 Wellcome Trust/CRUK Gurdon Institute, University of Cambridge, Cambridge CB2 1QN, UK

**Keywords:** Fibroblast lineages, Wounding, β-catenin

## Abstract

New hair follicles (HFs) do not form in adult mammalian skin unless epidermal Wnt signalling is activated genetically or within large wounds. To understand the postnatal loss of hair forming ability we monitored HF formation at small circular (2 mm) wound sites. At P2, new HFs formed in back skin, but HF formation was markedly decreased by P21. Neonatal tail also formed wound-associated HFs, albeit in smaller numbers. Postnatal loss of HF neogenesis did not correlate with wound closure rate but with a reduction in Lrig1-positive papillary fibroblasts in wounds. Comparative gene expression profiling of back and tail dermis at P1 and dorsal fibroblasts at P2 and P50 showed a correlation between loss of HF formation and decreased expression of genes associated with proliferation and Wnt/β-catenin activity. Between P2 and P50, fibroblast density declined throughout the dermis and clones of fibroblasts became more dispersed. This correlated with a decline in fibroblasts expressing a TOPGFP reporter of Wnt activation. Surprisingly, between P2 and P50 there was no difference in fibroblast proliferation at the wound site but Wnt signalling was highly upregulated in healing dermis of P21 compared with P2 mice. Postnatal β-catenin ablation in fibroblasts promoted HF regeneration in neonatal and adult mouse wounds, whereas β-catenin activation reduced HF regeneration in neonatal wounds. Our data support a model whereby postnatal loss of hair forming ability in wounds reflects elevated dermal Wnt/β-catenin activation in the wound bed, increasing the abundance of fibroblasts that are unable to induce HF formation.

## Abstract (precis)

**Summary:** Postnatal mouse skin exhibits a decline in its ability to regenerate hair follicles in the wound bed and this can be partially reversed by inhibiting dermal β-catenin activation.

## INTRODUCTION

Mammalian skin comprises two layers: the epidermis and dermis. The epidermis is made up of the interfollicular epidermis (IFE), the hair follicles (HFs), sweat glands and sebaceous glands and is maintained through the activity of distinct stem cell subpopulations (Watt, 2014). The underlying connective tissue, the dermis, is composed of distinct layers. The papillary (upper) dermis is the closest dermal layer to the epidermis and contains thin collagen fibres and a high density of fibroblasts. Below that lies the reticular (or lower) dermis, and beneath that lies the dermal white adipocyte (DWAT) layer, also known as the hypodermis (Driskell et al., 2014; Sorrell and Caplan, 2004). In addition, there are condensations of fibroblasts, called the dermal papilla (DP), at the base of the HFs, fibroblasts that ensheath the HF [dermal sheath (DS) cells] and smooth muscle cells in the arrector pili muscle (APM) attached to the HF (Driskell et al., 2011a; Sennett and Rendl, 2012). The epidermis and dermis form through highly coordinated epithelial-mesenchymal interactions during development (Driskell et al., 2011a; Fuchs and Horsley, 2008; Millar, 2002; Yang and Cotsarelis, 2010).

Skin fibroblasts from different anatomic sites have distinct and characteristic gene expression patterns (Rinn et al., 2006; Rinn et al., 2008). The differentially expressed genes are not only involved in defining cell positional identity, but are also associated with extracellular matrix (ECM) synthesis, lipid metabolism, proliferation, cell migration and fate determination (Chang et al., 2002). Positional differences in wound healing and regeneration ability have been reported, such that oral mucosa wounds heal faster with minimal scaring compared with cutaneous wounds (Chen et al., 2010).

At embryonic day (E) 12.5 of mouse development, dermal fibroblasts in back skin are capable of forming all the postnatal dermal compartments (Driskell et al., 2013). However, at E16.5 lineage restriction occurs, such that fibroblasts that are expressing Lrig1, Blimp1 (also known as Prdm1) or CD26 (also known as Dpp4) at that time point give rise to papillary dermis, including APM, DS and DP, while Dlk1-expressing fibroblasts give rise to the reticular dermis and DWAT (Driskell et al., 2013). The papillary lineage is required for new HF formation in skin reconstitution assays, while the lower lineage gives rise to the fibroblasts that mediate the initial phase of wound repair.

HF formation is normally restricted to embryonic development (Schmidt-Ullrich and Paus, 2005). However, new HFs can be induced in a range of adult mammals, including human, in the context of wound repair (Breedis, 1954; Kligman, 1959). In adult mice, new HFs form in the centre of very large (1 cm^2^) wounds as a result of epidermal Wnt signalling (Gay et al., 2013; Ito et al., 2007; Myung et al., 2013). Genetic activation of adult epidermal Wnt signalling via β-catenin can induce ectopic HF formation in the absence of wounding (Lo Celso et al., 2004; Silva-Vargas et al., 2005) and this correlates with expansion of the papillary dermal lineage (Driskell et al., 2013). Although new HFs do not normally form in small (≤8 mm) circular full-thickness wounds in adult mouse back skin, they can be induced if the number of papillary fibroblasts is expanded in response to epidermal Wnt signalling prior to wounding (Driskell et al., 2013).

Previous studies relating new HF formation to wound healing and the role of different fibroblast subpopulations led us to investigate why the ability to form new HFs is lost in postnatal life. We measured the time in postnatal life when HF formation no longer occurs in wounds and characterised the associated dermal changes. Our observations lead to the conclusion that loss of hair forming ability in adult wounds reflects increased recruitment of lower lineage fibroblasts at the expense of papillary fibroblasts, and is promoted by Wnt/β-catenin signalling in the wound dermis.

## RESULTS

### HF formation in wounds decreases with age and is location dependent

**Fig. 1.**
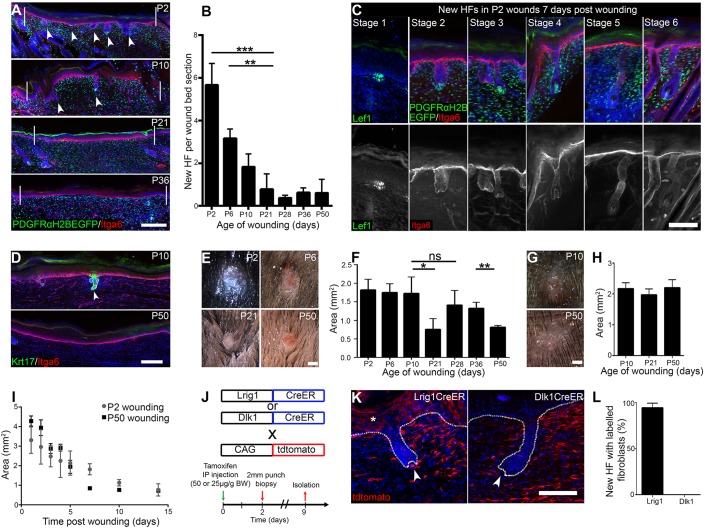
**Wound-induced new HF formation in neonatal back and tail skin.** (A) Histological analysis of 2 mm wounds performed in PDGFRaH2BeGFP mice postnatally by immunostaining for GFP (green) and Itga6 (red). Arrowheads indicate HFs within the wound bed and white lines demarcate the wound edges. (B) New HF quantitation per wound bed section at PW7. *n*=4 (P2, P21) or *n*=3 (other time points) biological replicates. (C) Analysis of different HF morphogenesis stages during HF formation in P2 wounds at PW7. Wound beds were immunostained for GFP (green) and Itga6 (red) or Lef1 (green) alone. (D) Wound-induced new HF formation in tail wounds. Tail wound beds were stained for Krt17 (green) and Itga6 (red) and quantified (see Table S1). Arrowhead indicates new HF. (E-H) Light micrographs and quantification of PW7 wound area in back (E,F) and tail (G,H) skin wounds made at the ages shown. (I) Wound area over time in neonatal and adult wounds as a surrogate for wound closure. *n*=6 biological replicates. (J-L) Lineage-tracing strategy (J), histological analysis of tdtomato^+^ fibroblasts (red) labelled with Lrig1CreER and Dlk1CreER (K), and quantitation (L) in PW7 wound beds from P2 wounded mice. (K) Dotted lines denote the epidermal-dermal boundary, arrowheads indicate developing DP and asterisk shows Lrig1CreER-labelled keratinocytes (Page et al., 2013). (L) Percentage of new HFs with attached tdtomato^+^ fibroblasts. Eight wound bed sections per mouse; *n*=4 Lrig1CreER, *n*=3 DLK1CreER. Nuclei are stained with DAPI (blue in A,C,D,K). Data shown are means±s.d. ns, not significant; *P<0.05, **P<0.005, ***P<0.0005. DP, dermal papilla; HF, hair follicle; IP, intraperitoneal. Scale bars: 100 µm in A,D; 50 µm in C,K; 50 mm in E,G.

To explore HF regeneration capacity, we made 2 mm full-thickness circular wounds on mouse back and tail skin of different ages. Seven days post wounding (PW7), new HFs had formed in the wound bed of P2 back skin, with an average of five new HFs per wound bed section (Fig. 1A,B). When P2 skin was wounded, all HF developmental stages (Müller-Röver et al., 2001) were present at PW7 (Fig. 1C), indicating that new HF formation was not a synchronised process. The more developed HFs (stages 4-6) tended to be at the wound edges, whereas placodes (stage 1) and stage 2-3 HFs were most abundant in the wound bed centre. The number of new HFs per wound did not change between PW7 and PW14 (data not shown), pointing to a limited, time-dependent window for HF regeneration during wound healing. New HF formation strongly declined with age, and after P21 only 0.8 new HFs per wound bed section formed. The decline was not correlated with the hair cycle (HC), since it occurred during the growth (anagen) phase of HF morphogenesis (P6, P10), the first postnatal telogen (resting phase; P21) and the second HC (P28-P50) (Fig. 1A,B).

P2 tails were too small to wound. However, when P10 tail was wounded new HFs were detected in the wounds, albeit fewer (Fig. 1D, Table S1) than in P10 back skin. This indicates that wound-induced HF regeneration potential is not restricted to one particular anatomical site. Tail HF formation also appeared to decline with age (Table S1). There was no correlation between wound area at PW7 and HF regeneration capacity in either tail or back skin (Fig. 1E-H). Back skin wound sizes at PW7 were significantly smaller when mice were wounded in telogen (P21, P50) than anagen (P2-P10, P28, P36), whereas in tail skin wound size did not change with age (Fig. 1F,H). Closure rates were similar in neonatal and adult wounds (Fig. 1I).

To establish the relative contributions of different fibroblast lineages to new HF formation, we labelled Lrig1^+^ or Dlk1^+^ cells via CreER-mediated recombination with tdtomato at P0, wounded back skin at P2 and analysed new HF formation at P9 (Fig. 1J,K). Cells of both the papillary (Lrig1^+^) and reticular (Dlk1^+^) lineages were present in the wound bed (Fig. 1K). The papillary fibroblast lineage was associated with the DS and DP of new HFs (eight wound bed sections analysed per mouse; *n*=4 mice), whereas the reticular lineage only contributed to the inter-HF dermis (eight wound bed sections analysed per mouse; *n*=3 mice) (Fig. 1K,L).

We conclude that neonatal skin of different anatomical sites is able to generate HFs at the site of small, full-thickness wounds and that papillary fibroblasts contribute to the new HF, DS and DP, as reported previously (Driskell et al., 2013). HF neogenesis is more pronounced in back than in tail skin, which might be due to differences in HF density of unwounded skin, and declines with age. HF formation is not dependent on the HF stage at which the wound is made and does not correlate with wound area at PW7.

### Changes in fibroblast gene expression with dermal maturation and location

**Fig. 2.**
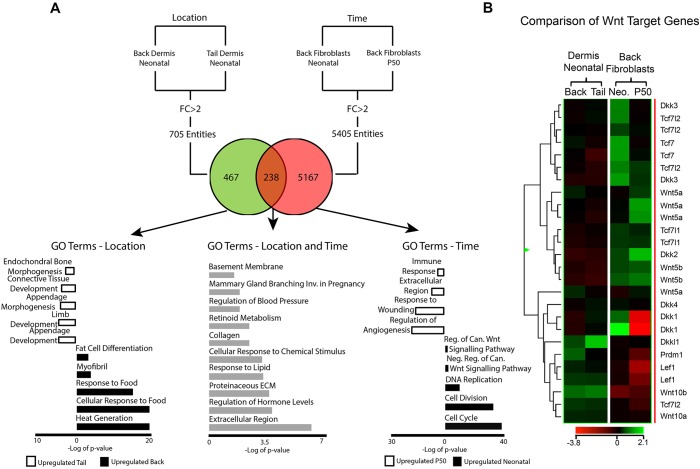
**Comparative gene expression analysis reveals differences associated with time and body location.** (A) Comparison of genes that are differentially expressed between P1 back and tail dermis (location) and between P2 and P50 dorsal fibroblasts (time). Representative GO term analysis of upregulated entities is shown. (B) Heatmap depicting the differentially expressed Wnt target genes among the four samples analysed. Can., canonical; FC, arbitrary fold change; Inv., involved; Neg., negative; Neo., neonatal; Reg., regulation.

To uncover potential explanations for the differences in HF formation at different ages and body sites, we performed gene expression profiling of neonatal (P1) back and tail dermis (GSE83117, Table S2) and compared the results with a published dataset of back skin dermal fibroblasts isolated from neonatal (P2) and adult (P50) mice (Collins et al., 2011). We found 705 entities that were differentially expressed (≥2-fold) between neonatal back and tail dermis, and 5405 entities that were differentially expressed in neonatal and adult back skin fibroblasts (Fig. 2A). Only 238 entities overlapped between the two datasets, reflecting genes that changed with both time and location. Therefore, the majority of differences in gene expression are between neonatal and adult dermis rather than between neonatal dermis in different anatomical locations.

Gene ontology (GO) analysis of entities differentially represented between neonatal tail and back dermis included the GO terms ‘connective tissue’ and ‘appendage development’, which were more highly represented in the tail. ‘Fat metabolism processes’ was more highly represented in back dermis, consistent with the lack of DWAT in tail skin.

GO terms for entities that changed in back skin between P2 and P50 included ‘cell division’, ‘cell cycle’ and ‘DNA replication’, all of which were significantly downregulated during postnatal skin maturation. By contrast, there was a higher representation of entities associated with immune response, angiogenesis, ECM and wound healing in adult back fibroblasts. Additionally, many Wnt pathway components were downregulated with age, whereas there was little differential expression of Wnt pathway genes between neonatal back and tail dermis (Fig. 2B).

Taken together, the microarray comparisons of RNA isolated from the dermis at different times and locations revealed that the positional identity of fibroblasts (back versus tail) affected only a small number of genes, whereas postnatal dermal maturation triggered changes in multiple genes, including the downregulation of genes associated with cell proliferation and Wnt signalling.

### Postnatal dermal expansion occurs with minimal fibroblast proliferation

**Fig. 3.**
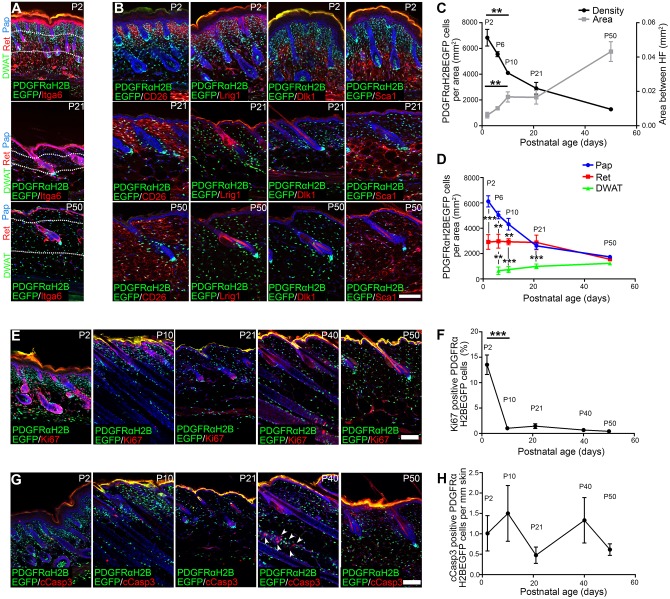
**Changes in fibroblast density, marker expression, proliferation and apoptosis in postnatal back skin.** (A-D) Fibroblast density and marker expression analysis. Immunostaining for Itga6 (A) and CD26, Lrig1, Dlk1 and Sca1 (red) (B) in PDGFRaH2BeGFP (green) skin. White dotted lines (A) mark dermis layer boundaries. (C) Mean dermal area between adjacent HFs (right *y*-axis) and total fibroblast density per mm^2^ of dermis (left *y*-axis) over time. (D) Fibroblast density in dermis layers indicated in A. (E,F) Ki67 staining (red) of whole-mounts (E) and quantification (F) of percentage Ki67^+^ PDGFRaH2BeGFP cells (green). (G,H) Cleaved caspase 3 (cCasp3) staining (red) of whole-mounts (G) and quantification (H) of percentage cCasp3^+^ PDGFRaH2BeGFP cells (green). Note that most apoptotic cells in the skin are observed in the interfollicular epidermis (IFE) and HF keratinocytes (arrowheads in G). Nuclei were labelled with DAPI (blue in A,B,E,G). Pap, papillary dermis; Ret, reticular dermis; DWAT, dermal white adipose tissue. Data shown are means±s.d. *n*=3 biological replicates per time point (C,D,F,H). **P<0.005, ***P<0.0005. Scale bars: 100 µm.

To evaluate changes in postnatal dermis that correlate with loss of HF forming ability, we labelled the dermis of PDGFRaH2BeGFP mice (with a histone H2B-eGFP reporter knocked into the *Pdgfra* locus) for markers that distinguish different fibroblast subpopulations at P2 (Driskell et al., 2013) (Fig. 3A,B). Quantitation of total dermal fibroblasts, based on the expression of nuclear EGFP, showed a striking reduction in fibroblast density between P2 and P10, with further reductions at P21 and P50 (Fig. 3C). By contrast, between P2 and P50 the area between adjacent HFs increased markedly, reflecting dermal expansion (Fig. 3C). When we scored cell density separately in the papillary, reticular and DWAT layers (Fig. 3D), we found that papillary dermis had the highest cell density at P2 and showed a marked decrease at P21. However, between P21 and P50 papillary and reticular cell density both decreased. By contrast, DWAT cell density marginally increased with age, and at P50 the density in all three dermal layers was similar (Fig. 3A,D). During skin maturation there were also major changes in expression of the P2 markers of papillary (CD26^+^, Lrig1^+^) and reticular/DWAT (Dlk1^+/−^, Sca1^+^) dermis, as previously reported (Driskell et al., 2013). CD26 and Sca1 (also known as Ly6a) expression extended throughout the dermis with age, whereas Lrig1 and Dlk1 were strongly downregulated (Fig. 3B).

To investigate whether the dermal changes correlated with fibroblast proliferation and apoptosis, we stained PDGFRaH2BeGFP back skin whole-mounts for Ki67 and cleaved caspase 3 (cCasp3) (Fig. 3E-H). We observed a strong reduction in Ki67^+^ fibroblasts between P2 and P10 (Fig. 3E,F), and proliferation remained low with increasing age. Very few cCasp3^+^ fibroblasts were detected at any age (Fig. 3G,H), while apoptosis in the epidermis was HC dependent, as reported previously (Lindner et al., 1997).

We conclude that during dermal maturation the area between HFs increases, while fibroblast density decreases. The most pronounced decrease in cell density is in the papillary layer, coinciding with the loss of HF neogenesis in wounds. The decrease in dermal cell density does not correlate with increased apoptosis, and after P2 there is very little fibroblast proliferation, consistent with the microarray analysis (Fig. 2A).

### Clonal analysis of fibroblasts during dermal maturation

**Fig. 4.**
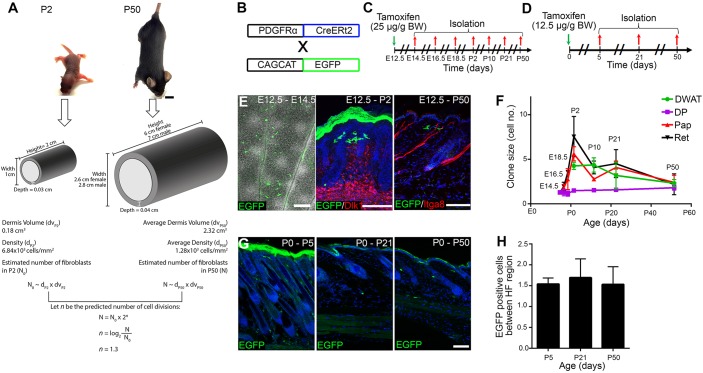
**Estimation of cellular replication during dermal maturation and clonal analysis of PDGFRaCreERt2-positive cells.** (A) Predicted number of dermal fibroblast divisions (trunk skin) during the transition from neonatal (P2) to adult (P50) mouse. Height, length and dermis diameter were measured (*n*=3 mice per time point and gender) and the dermis volume was estimated by representing the mouse trunk as a cylinder. Cell densities were obtained from Fig. 3C and the cell number at P2 (*N*_0_) and P50 (*N*) were estimated by multiplying cell density and dermis volume. The predicted cell division rate (*n*=1.3) is calculated by the log_2_ of the *N*/*N*_0_ ratio. All raw data for the calculations are shown in Table S3. (B-H) Clonal analysis of PDGFRaCreERt2^+^ cells. (B) Breeding strategy for E-H. (C) Labelling strategy for E,F. (D) Labelling strategy for G,H. (E) Immunostaining of GFP^+^ fibroblasts (green) co-stained for Dlk1 or Itga8 (red) or overlaid with the brightfield image. (F) Quantitation of GFP^+^ cells per clone per compartment. *n*=3 biological replicates per time point. Cells were considered clonally related if they were contained within a total dermal area of 260 µm diameter (Driskell et al., 2013). (G) Immunostaining of GFP^+^ fibroblasts. (H) Quantitation of GFP^+^ fibroblasts in the area between adjacent HFs. *n*=3 biological replicates per time point. Nuclei were labelled with DAPI (blue in E,G). Data shown are means±s.d. Scale bars: 1 cm in A; 100 µm in E (middle and right), G; 500 µm in E (left).

To gain more insight into the changes in fibroblast number and distribution during dermal maturation we first used our experimental measurements (Fig. 3C, Table S3) to model the number of cell divisions between P2 and P50 (Fig. 4A). By calculating mouse body size at each stage and modelling the body as a cylinder, we calculated that dermal volume increases 13-fold from 0.18 cm^3^ (P2 mouse) to 2.32 cm^3^ (average between P50 male and female mice). Combining this with the fibroblast density measurements (Fig. 3C), we predicted that on average only 1.3 cell divisions occur in PDGFRa (Pdgfrα)^+^ fibroblasts between P2 and P50 (Fig. 4A). This is consistent with the low number of proliferating cells observed experimentally (Fig. 3E,F). From here we could further predict that individual fibroblasts labelled at E12.5 would initially form clones of increasing cell number, but after P2 clone size would seem to decrease as clonally related cells became distributed over an increasing area of dermis.

To test our hypothesis we performed clonal analysis, defining the maximum diameter occupied by clonally related cells as 260±50 µm, as described previously (Driskell et al., 2013). Fibroblasts in bi-transgenic PDGFRaCreERt2×CAGCATeGFP mice were labelled with a low tamoxifen dose, which resulted in 1-2 PDGFRa^+^ cells being labelled in the dermis between adjacent HFs (inter-HF dermal area) (Fig. 4B-D). When fibroblasts were labelled at E12.5 they gave rise to clones of 4-8 cells spanning the papillary and reticular dermis and DWAT at P2 (Fig. 4E,F). Thereafter, the number of cells per clone decreased in the papillary and reticular dermis and DWAT, but not in the DP, consistent with DP cells forming a condensate (Kaushal et al., 2015; Tobin et al., 2003) (Fig. 4F). When clonal labelling was performed in bi-transgenic mice at P0 (Fig. 4D,G), there was no subsequent increase in EGFP^+^ cells (Fig. 4H), consistent with the very low proliferation rate in postnatal skin (Collins et al., 2011) (Fig. 3F).

We conclude that, consistent with the postnatal increase in dermal volume occurring with very little fibroblast proliferation, clonally related cells become progressively separated from one another.

### Postnatal dermal maturation coincides with a reduction in dermal β-catenin signalling

**Fig. 5.**
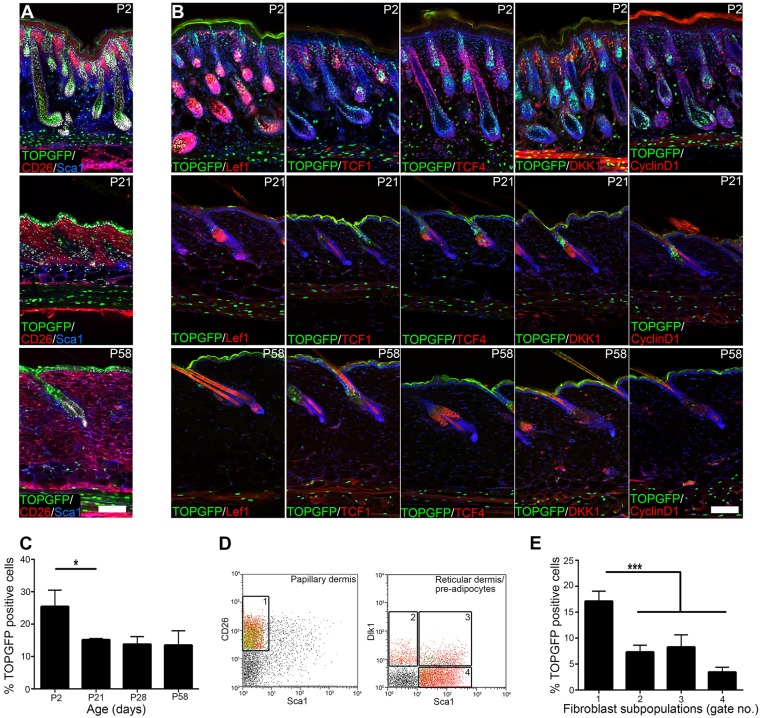
**Dermal Wnt signalling activity.** (A,B) Immunostaining for TOPGFP signal (green) and the markers indicated: CD26 (red) and Sca1 (blue) in A; Lef1, TCF1, TCF4, Dkk1 and cyclin D1 (red) in B. Nuclei were labelled with DAPI (white in A, blue in B). (C) Quantitation of percentage TOPGFP^+^ dermal fibroblasts determined from histological sections. *n*=3 biological replicates per time point. (D,E) Flow cytometry analysis of TOPGFP expression in P2 fibroblast subpopulations. Gate 1, CD26^+^ Sca1^−^ (papillary fibroblasts); gate 2, Dlk1^+^ Sca1^−^ (reticular fibroblasts); gate 3, Dlk1^+^ Sca1^+^; gate 4, Dlk1^−^ Sca1^+^ (cells with adipogenic potential). C is representative of *n*=3 biological replicates shown in D. Data shown are means±s.d. *P<0.05, ***P<0.0005. Scale bars: 50 µm.

In addition to predicting a reduction in fibroblast proliferation during dermal maturation, the microarray comparison suggested a significant decrease in Wnt/β-catenin signalling. As a readout of dermal Wnt signalling we examined the TOPGFP reporter mouse, in which H2BeGFP is expressed under the control of multiple Lef1/TCF binding sites (Ferrer-Vaquer et al., 2010) (Fig. 5). As previously reported, at E12.5 Wnt/β-catenin signalling is highly active in most fibroblasts (Chen et al., 2012; data not shown). TOPGFP^+^ fibroblasts were abundant at P2, but declined significantly by P21 (Fig. 5A-C). At P2 the number of cells with detectable nuclear TOPGFP, as evaluated by flow cytometry (Fig. 5D,E), was 17.1% within the papillary (CD26^+^ Sca1^−^) population, compared with 7.2% in the reticular (Dlk1^+^ Sca1^−^) population and 11.8% in fibroblasts with adipogenic potential (8.3% Dlk1^+^ Sca1^+^; 3.5% Dlk1^−^ Sca1^+^) (Driskell et al., 2013; Festa et al., 2011). This was confirmed by immunofluorescent staining, since at P2 the upper dermis (enriched for papillary Lrig1^+^ CD26^+^ fibroblasts) contained more TOPGFP^+^ cells than the lower dermis (Dlk1^+^ Sca1^+^) (Fig. 5A).

In line with the microarray analysis, proteins encoded by Wnt target genes such as *Lef1*, *Dkk1* and cyclin D1 were highly expressed in the upper dermis at P2 (Fig. 5B). While most TOPGFP^+^ cells in the upper dermis showed high nuclear Lef1 levels, in the lower dermis TCF4 (also known as Tcf7l2) was nuclear in cells that were TOPGFP^+^. By contrast, nuclear TCF1 (also known as Tcf7) was mainly confined to the papillary fibroblast lineage. These observations are consistent with gene expression profiles of neonatal fibroblasts, showing that papillary fibroblasts have an active Wnt signalling signature, but also highlight that Wnt signalling can simultaneously occur in the lower dermis (Driskell et al., 2013; Mastrogiannaki et al., 2016).

In adult skin there were fewer TOPGFP^+^ cells throughout the dermis, with adipocytes and APM cells showing the highest TOPGFP signal (Fig. 5A-C). The decrease in dermal TOPGFP activity was associated with a decrease in the expression of all TCFs as well as all analysed Wnt target genes (Fig. 5B).

We conclude that postnatal dermal maturation is associated with a reduction in Wnt/β-catenin signalling. Additionally, there is differential signalling activity in different fibroblast subpopulations.

### Wound-induced dermal β-catenin activation in fibroblasts increases with age

**Fig. 6.**
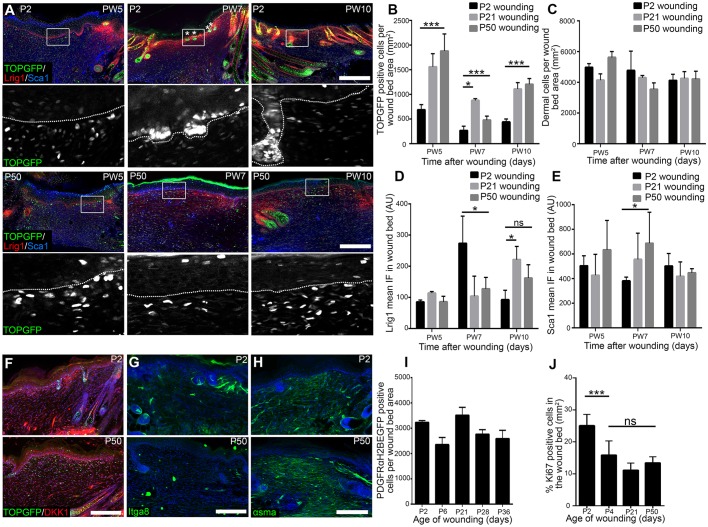
**Analysis of neonatal and adult wound beds.** (A,B) TOPGFP expression in fibroblasts of PW5, PW7 and PW10 wound beds of mice wounded at neonatal (P2) and adult (P21 and P50) stages. Wound beds were immunostained for GFP (green), Lrig1 (red) and Sca1 (blue) (A). TOPGFP expression in the boxed areas is shown at higher magnification beneath. Placodes are marked with asterisks and dotted lines indicate the basement membrane. (B) Quantitation of GFP^+^ cells in wound beds at the indicated time points. *n*=4 biological replicates, except *n*=3 for P21 and P50 at PW7. (C) Dermal cells per mm^2^ wound bed area over time in adult and neonatal wounds. *n*=4 biological replicates. (D,E) Mean immunofluorescence quantification in the neonatal (P2) and adult (P21, P50) wound beds over time for Lrig1 (D) and Sca1 (E). *n*=4 biological replicates per time point. (F) Dkk1 expression (red) in TOPGFP (green) P2 and P50 wound beds at PW7. (G,H) PW7 wound beds after wounding at the indicated time point, immunostained for Itga8 (green, G) and αSma (green, H). (I) Quantitation of the density of PDGFRaH2BeGFP-expressing cells in PW7 wound beds wounded at the indicated time points. *n*=3 biological replicates per time point. (J) Quantitation of Ki67^+^ cells in the wound beds after wounding at the indicated time points. *n*=8 (P2), *n*=6 (P4), *n*=4 (P21), *n*=5 (P50) biological replicates. Nuclei were labelled with DAPI (white in A; blue in F-H). Data shown are means±s.d. ns, not significant; *P<0.05, ***P<0.0005. AU, arbitrary units; IF, immunofluorescence. Scale bars: 100 µm.

To examine whether the age-associated fibroblast density changes and Wnt/β-catenin signalling in unwounded skin were mirrored by changes during wound healing, we investigated β-catenin activity in wound beds of neonatal and adult TOPGFP mice at PW5, PW7 and PW10. Surprisingly, there was strong dermal Wnt/β-catenin activation in adult P21 and P50 wound beds, whereas neonatal P2 wound beds had fewer TOPGFP^+^ cells at all time points (Fig. 6A,B). By contrast, the epidermis showed similar TOPGFP activation in the wound bed regardless of age at all time points, except for local upregulation in the placodes of new HFs (Fig. 6A).

During embryogenesis the Wnt signalling inhibitor Dkk1 is highly expressed by dermal fibroblasts, creating a permissive environment for HF formation by locally suppressing Wnt/β-catenin signalling (Andl et al., 2002; Sick et al., 2006). P2 wound beds had strong Dkk1 expression in the upper dermis, especially surrounding the developing HF (Fig. 6F). Dkk1 expression was strongly reduced in adult wound beds (Fig. 6F), correlating with the increased number of TOPGFP^+^ cells (Fig. 6A,B).

Although the total dermal cell density in wounds did not change significantly with age (Fig. 6C,I), the number of cells expressing papillary (Lrig1) and reticular (Sca1) markers changed as a function of age at wounding and the number of days post wounding (Fig. 6D,E) (Driskell et al., 2013). In P50 mice, more Sca1^+^ cells were present in the wound bed 7 days after wounding than in P2 mice, while in P2 wounds Lrig1^+^ cells predominated at PW7, particularly close to the basement membrane when new HFs form (Fig. 6A,D,E). In adult wound beds the number of Lrig1^+^ cells increased at PW10 when wound-induced HF neogenesis is not observed (Fig. 6D). Similarly, there were more Itga8^+^ cells (a previously described marker of papillary fibroblasts, DP and APM cells in P2 skin; Driskell et al., 2013) in neonatal than in adult wound beds at PW7 (Fig. 6G). By contrast, adult wounds contained a higher proportion of cells that expressed α-smooth muscle actin (αSma) at PW7 (Fig. 6H), which we have previously shown, by lineage tracing, to derive from the lower dermal lineage (Driskell et al., 2013).

Following P2 wounding there were significantly more proliferating (Ki67^+^) cells in PW7 wound beds than at P4 (Fig. 6J). However, there was no significant difference in proliferation in the wound beds of mice wounded at P4 and P50, even though HF neogenesis declined during that period (Fig. 1B). Fibroblast density within neonatal and adult wound beds did not differ significantly at PW7 (Fig. 6I).

We conclude that the loss of HF forming ability correlates with an increase in dermal Wnt/β-catenin signalling, a decrease in the proportion of cells expressing papillary markers and an increase in αSma^+^ cells in the wound bed at PW7, when new HFs are able to form. However, wound beds that differ in their ability to support HF regeneration do not differ in fibroblast proliferation and density.

### Promotion of HF formation in adult wounds by inhibition of Wnt/β-catenin signalling

**Fig. 7.**
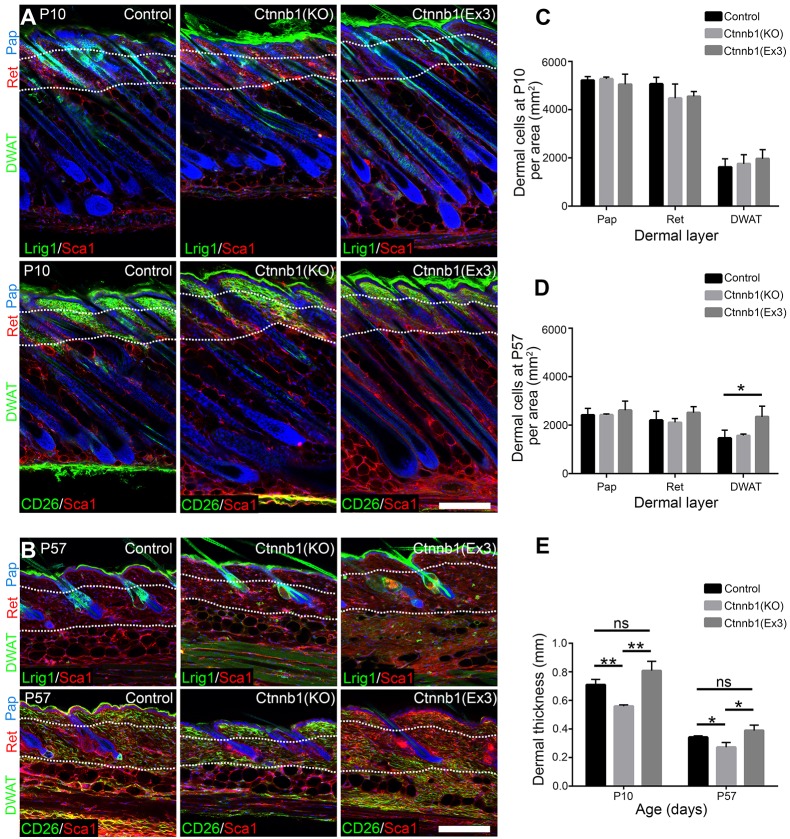
**Effect of postnatal dermal β-catenin ablation or activation on skin homeostasis.** (A,B) Immunostaining of Lrig1 (green, top rows), CD26 (green, bottom rows) and Sca1 (red) in neonatal (P10) and adult (P57) whole-mount sections of wild-type mice (control) or following β-catenin (*Ctnnb1*) ablation [Ctnnb1(KO)] or activation [Ctnnb1(Ex3)], as shown in Fig. 9A. White dotted lines indicate dermis layer boundaries. (C,D) Quantification of cell densities in the indicated dermis layers in A and B in neonatal (P10, C) and adult (P57, D) whole-mount sections. *n*=6 whole-mount sections per mouse, *n*=3 biological replicates per genotype and time point. (E) Quantification of dermal thickness at the indicated time points. *n*=5 biological replicates per genotype and time point. Nuclei were labelled with DAPI (blue in A,B). Data shown are means±s.d. ns, not significant; *P<0.05, **P<0.005. Scale bars: 100 µm.

To determine whether altering dermal Wnt/β-catenin signalling would affect HF formation in the wound bed, we specifically deleted or activated β-catenin in fibroblasts by crossing *Ctnnb1*^flox/flox^ or *Ctnnb1*^+/flox(ex3)^ with PDGFRaCreERt2×tdtomato mice and applying tamoxifen after birth (P0 and P3) (Mastrogiannaki et al., 2016). Both deletion and activation of β-catenin resulted in mice that were viable but smaller, with a shorter tail than controls (data not shown), effects that probably reflect PDGFRaCre activity in tissues such as the CNS, bone marrow and developing bone (Betsholtz, 1995). Deletion or activation of β-catenin did not alter fibroblast density in the papillary and reticular layers, nor did it affect the expression of papillary and reticular markers in unwounded neonatal and adult dermis (Fig. 7A-D). There was no effect on APM number or size (data not shown). However, whereas at P10 the DWAT was normal, by P57 dermal β-catenin stabilisation led to a reduction in adipocytes and the accumulation of scattered fibrotic patches, resulting in an increase in DWAT cell density and total dermal thickness, as previously reported (Mastrogiannaki et al., 2016; Hamburg-Shields et al., 2015; Hamburg and Atit, 2012) (Fig. 7A-E). By contrast, in β-catenin-deleted mice dermal thickness was slightly reduced (Fig. 7E).

**Fig. 8.**
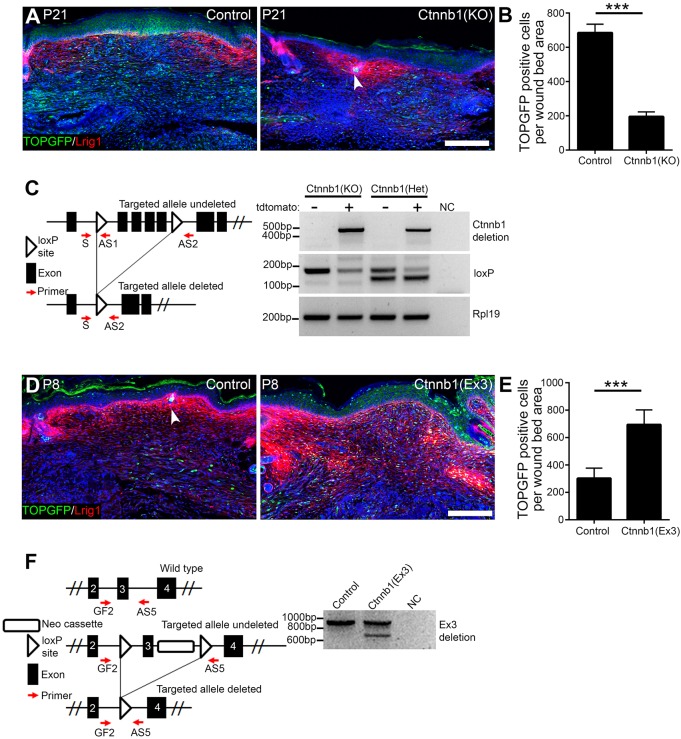
**TOPGFP activity and β-catenin deletion efficiency in dermal fibroblasts.** (A) TOPGFP activity (green) with Lrig1 immunostaining (red) in P21 wounds at PW7 of wild-type (control) and β-catenin-deleted [Ctnnb1(KO)] mice. (B) Quantitation of GFP^+^ cells in PW7 wound beds. *n*=6 whole-mount sections per mouse; *n*=4 control, *n*=2 Ctnnb1(KO) biological replicates. (C) Schematic representation of the undeleted and deleted β-catenin targeted allele. DNA from sorted tdtomato-positive (+) and tdtomato-negative (−) cells was tested for β-catenin deletion (primers indicated by red arrows). Ctnnb1(KO) shows a 480 bp band indicating β-catenin deletion and loss of the 180 bp loxP PCR product, whereas undeleted cells (tdtomato^−^) generate only the 180 bp loxP PCR product. In heterozygous mice [Ctnnb1(Het)] the untargeted allele amplifies as a 150 bp band owing to the missing loxP site. *Rpl19* PCR was used as loading control. (D) TOPGFP activity (green) with Lrig1 immunostaining (red) in P8 wounds at PW7 of wild-type (control) and β-catenin-stabilised [Ctnnb1(Ex3)] mice. (E) Quantitation of GFP^+^ cells in PW7 wound beds. *n*=6 whole-mount sections per mouse; *n*=4 biological replicates per genotype. (F) Schematic representation of the wild-type, undeleted and deleted targeted exon 3 allele for β-catenin stabilisation. DNA isolated from whole-mount sections was tested for β-catenin exon 3 deletion (primers indicated by red arrows). The wild-type allele amplifies as a 900 bp band, the undeleted targeted allele gives no product and the deleted targeted allele amplifies as a 700 bp band. Note that Ctnnb1(Ex3) transgenic is kept heterozygous, and thus PCR shows efficient recombination. NC, negative control (no DNA). (A,D) Nuclei were labelled with DAPI (blue) and arrowheads indicate new HFs. Data shown are means±s.d. ***P<0.0005. Scale bars: 100 µm.

To confirm that dermal β-catenin modulation had indeed been achieved, we examined TOPGFP activity (Fig. 8). As expected, β-catenin deletion substantially reduced Wnt reporter activity in adult P21 wound beds (Fig. 8A,B), where Wnt/β-catenin signalling is high in control mice (Fig. 6A,B). Conversely, β-catenin stabilisation increased the number of TOPGFP^+^ cells in neonatal P8 wounds (Fig. 8D,E), where Wnt/β-catenin signalling is low in controls (Fig. 6A,B). In addition, we sorted tdtomato^+^ cells or used whole-mount tissue for PCR analysis, confirming efficient deletion of the floxed exons in both *Ctnnb1*^flox/flox^ and *Ctnnb1*^+/flox(ex3)^ cells (Fig. 8C,F); this is comparable to results reported in other skin studies (see Enshell-Seijffers et al., 2010a).

**Fig. 9.**
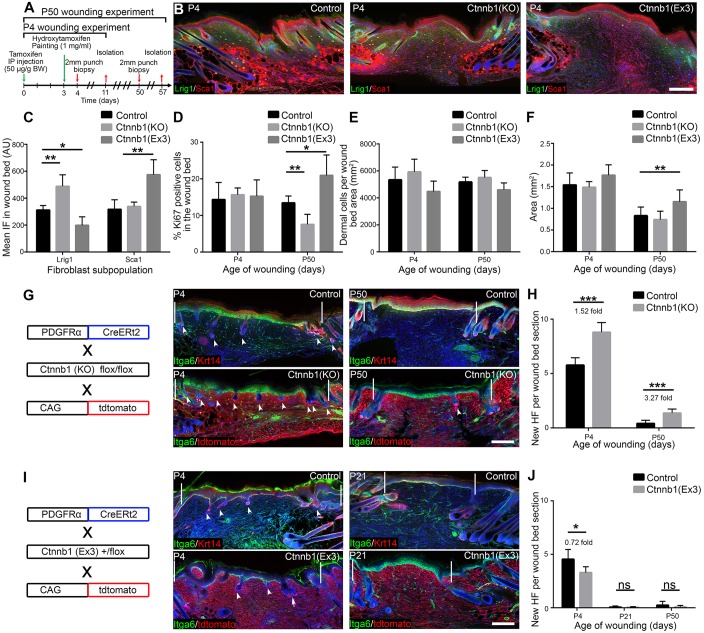
**Effect of β-catenin ablation or activation on HF formation in wound beds.** (A) Experimental design. (B,C) Immunostaining analysis (B) and quantification (C) for Lrig1 (green) and Sca1 (red) in PW7 wound beds of wild-type mice (control) or following β-catenin ablation [Ctnnb1(KO)] or activation [Ctnnb1(Ex3)] wounded at P4. *n*=3 whole-mount sections per mouse; *n*=5 biological replicates per genotype. (D-F) Quantification of Ki67^+^ cells in the wound bed area (D), dermal cells in the wound bed (E) and wound bed area (F) at PW7 of wild-type mice or following β-catenin ablation or activation. Mice were wounded at the indicated time points. (D) *n*=5 for P50 control, Ctnnb1(KO), *n*=7 for P4 control, *n*=4 for P4 Ctnnb1(KO), Ctnnb1 (Ex3), P50 Ctnnb1(Ex3); (E) *n*=5; (F) *n*=6 biological replicates. (G,I) Immunostaining of new HFs in PW7 wound beds of mice following β-catenin ablation (G) or activation (I) in postnatal fibroblasts. Wound beds were immunostained for Krt14 (red), Itga6 (green) or tdtomato (red) as indicated. White lines demarcate wound edges; arrowheads indicate new HFs and arrow indicates an enlarged new HF. (H,J) Quantitation of HF formation in wound beds shown in G and I. (H) *n*=8 for P4 control, P50 Ctnnb1(KO), *n*=6 for P4 Ctnnb1(KO), *n*=9 for P50 control; (J) *n*=9 for P4 control, *n*=4 for P4 Ctnnb1(Ex3), P50 control, Ctnnb1(Ex3), *n*=3 for P21 control, Ctnnb1(Ex3) biological replicates. Nuclei were labelled with DAPI (blue in B,G,I). Data shown are means±s.d. ns, not significant; *P<0.05, **P<0.005, ***P<0.0005. AU, arbitrary unit; BW, body weight; IF, immunofluorescence. Scale bars: 100 µm.

Next we wounded the skin at P4 (the earliest possible time point after β-catenin modulation) or P50 (adult) and analysed the wound bed at PW7 (Fig. 9A). In P4 wounds, dermal β-catenin activation led to an increase in Sca1^+^ cells in the wound bed, whereas more Lrig1^+^ cells were present after dermal β-catenin deletion at PW7 (Fig. 9B,C). Although proliferation (Ki67^+^) in adult wound beds was significantly decreased in β-catenin-deleted dermis and increased in β-catenin-activated dermis (Fig. 9D) (Beyer et al., 2012; Chen et al., 2012), dermal cell density was not significantly affected at PW7 (Fig. 9E). In addition, wound size was slightly increased in P50 β-catenin-activated mice (Fig. 9F) and, as previously shown, β-catenin modulation did not affect wound closure (Cheon et al., 2006).

Intriguingly, HF formation in the wound bed was significantly increased (1.52-fold) on dermal deletion of β-catenin (Fig. 9G,H), whereas β-catenin stabilisation inhibited HF formation by 30% in P4 wounds (Fig. 9I,J). We occasionally observed enlarged HFs in the wound bed upon β-catenin stabilisation (Fig. 9I, arrow), as has been reported when dermal β-catenin is stabilised during embryonic development (Chen et al., 2012). When β-catenin-deleted mice were wounded at P50, HF regeneration increased 3.27-fold (Fig. 9H). By contrast, β-catenin stabilisation in fibroblasts did not affect HF regeneration at P21 or P50 (Fig. 9I,J).

We conclude that, consistent with the observed reduction in dermal Wnt signalling with age (Fig. 5), β-catenin is not essential for fibroblasts to contribute to dermal maturation. Wnt/β-catenin signalling inhibition in fibroblasts promotes HF regeneration during wound healing, which correlates with a reduction in the number of cells expressing markers of reticular fibroblasts and an increase in cells expressing papillary fibroblast markers.

## DISCUSSION

It has previously been reported that new HF formation ceases after E19.5 of mouse development (Colwell et al., 2006; Martin et al., 2003; Shaw and Martin, 2009). Here we demonstrate that neonatal mouse skin is capable of forming new HFs during wound healing and that this ability is largely lost within the first 3 weeks after birth. The loss of HF ability correlates with a decline in papillary fibroblast density and dermal β-catenin signalling in undamaged skin. However, on wounding, dermal β-catenin activity is upregulated to a greater extent in older than in neonatal wounds and this is linked to a greater proportion of fibroblasts expressing reticular markers, consistent with previous lineage-tracing studies that showed these cells lack HF inducing ability (Driskell et al., 2013). By ablating β-catenin in PDGFRa^+^ cells we were able to partially restore HF formation in wounded older skin. Our results are consistent with an earlier report showing that during wound repair β-catenin signalling is strongly upregulated in the fibroblasts of adult mice (Cheon et al., 2006). It is notable that the loss of hair forming capacity in adult skin does not reflect a change in the ability of the epidermis to form new HFs, since ectopic HFs can be induced in unwounded and wounded skin by epidermal β-catenin activation (Deschene et al., 2014; Lo Celso et al., 2004; Ito et al., 2007; Silva-Vargas et al., 2005).

It is interesting to speculate that one reason why P2 skin can regenerate HFs whereas P21 skin cannot is that from P5 onwards many of the papillary fibroblasts are recruited to form the APM and this may render them unable to contribute to restoring the papillary dermis (Driskell et al., 2013). However, altered dermal β-catenin activity did not affect the number or size of individual APM. There is no evidence that existing DP cells contribute significantly to wound repair in adult skin (Johnston et al., 2013; Kaushal et al., 2015). Nevertheless, new DP can be induced upon epidermal β-catenin activation (Silva-Vargas et al., 2005). Neonatal tail skin was able to form new HFs in wounds and this is consistent with the lack of differential Wnt gene expression between tail and back (Fig. 2). We speculate that there are fewer wound-associated HFs in neonatal tail skin because of the lower density of HFs in unwounded tail or the lack of papillary progenitors of the APM.

The importance of Wnt signalling in skin fibroblasts has been shown in a number of different contexts. In early embryogenesis, before E16.5, β-catenin expression in fibroblasts is required for the formation of the dorsal and ventral dermis, while ectopic β-catenin activation increases fibroblast density and disturbs epidermal morphogenesis (Atit et al., 2006; Chen et al., 2012; Ohtola et al., 2008). In late embryonic dermis, when fibroblast lineages have been established, altered β-catenin activity in the DP causes defects in HF growth, cycling and pigmentation but does not cause HF loss (Enshell-Seijffers et al., 2010a,b). By contrast, β-catenin activation in postnatal dermal fibroblasts causes fibrosis in the adipocyte layer (Mastrogiannaki et al., 2016; Hamburg and Atit, 2012; Hamburg-Shields et al., 2015), supporting our observations in older unwounded skin (Fig. 7B). This corroborates the idea that β-catenin can be simultaneously active in different fibroblast lineages performing distinct functions. Interestingly, during wound repair ectopic β-catenin activation throughout the skin leads to increased wound size, fibroblast proliferation and fibrosis, while β-catenin deletion decreases proliferation and wound size (Fig. 9D,E) (Beyer et al., 2012; Cheon et al., 2006).

It is interesting that postnatal mouse growth does not correlate with an increase in fibroblast proliferation within the dermis (Collins et al., 2011, 2012), and our clonal analysis is consistent with a model whereby fibroblasts within a clone become separated from one another through ECM deposition. Fibroblast β-catenin activation can stimulate ECM production (Hamburg-Shields et al., 2015), and although this could play a role in dermal wound repair it is unlikely to be the major mediator of postnatal dermal expansion, since signalling is downregulated postnatally.

We conclude that postnatal dermal maturation facilitates rapid wound repair through recruitment of lower dermal fibroblasts but that this comes at the expense of the ability to regenerate HFs at the wound site.

## MATERIALS AND METHODS

### Transgenic mice

All animal experiments were subject to local ethical approval and performed under the terms of a UK government Home Office license. All mice were outbred on a C57BL6/CBA background and male and female mice were used in experiments that included PDGFRaCreERt2 (Rivers et al., 2008), Lrig1CreERt2-IRES-GFP (Page et al., 2013), Dlk1CreERt2 (Driskell et al., 2013), ROSAfl-stopfl-tdTomato (Jackson Laboratories, 007905), CAGCATeGFP (Kawamoto et al., 2000), TopH2BeGFP (Ferrer-Vaquer et al., 2010), PDGFRaH2BeGFP (Hamilton et al., 2003), β-catenin floxed (*Ctnnb1*^flox/flox^) (Huelsken et al., 2001) and β-catenin exon 3 deletion [*Ctnnb1*^+/flox(ex3)^] (Harada et al., 1999) mice. To perform lineage tracing or modulate β-catenin activity in fibroblasts, PDGFRaCreERt2 mice were bred to the appropriate floxed mice and treated with tamoxifen.

### Wounding experiments

Analgesic EMLA cream 5% (AstraZeneca) was applied to the skin for 10 min before mice were anaesthetised using isoflurane (Cp-pharma). A 2 mm diameter punch biopsy (Stiefel) was used to make a full-thickness wound in the central back skin or tail base at the indicated time points. If necessary, the hair on the back was clipped prior to wounding. When neonatal mice (P2-P10) were wounded, all litter pups were wounded and housed with their mother until weaning age. Wound closure was quantitated by comparing digital wound bed photos taken at different times after wounding.

### Clonal analysis

CAGCATeGFP mice were crossed with PDGFRaCreERt2 mice. Cre was induced at E12.5 by injecting pregnant females with 25 µg tamoxifen/g body weight, which we have previously shown labels fewer than 1% of all skin fibroblasts (Driskell et al., 2013). At E14.5 GFP^+^ cells were analysed in *n*=3 horizontal whole-mounts per embryo (*n*=3 embryos per time point) by confocal microscopy. All labelled cells within a 260±50 µm radius (Driskell et al., 2013) were scored as being clonally related. For postnatal clonal analysis neonatal mice (P0) were injected with 12.5 µg tamoxifen/g body weight, labelling 1-2 cells per HF region, i.e. the area of dermis between adjacent HFs, and analysed at different time points. All clones in *n*=3 whole-mounts per mouse (*n*=3 mice per time point) were analysed.

### Immunostaining

Immunostaining was performed on 60 µm cryosections stained as horizontal whole-mounts, as previously described (Driskell et al., 2013, 2011b). Briefly, skin was fixed in 4% PFA for 15-30 min, washed with PBS and embedded in OCT (Sakura Finetek). Sections (60 µm) were placed in PBS to dissolve the OCT. Sections were stained in 300-500 µl PB buffer (PBS containing 0.5% skimmed milk, 0.25% cold water fish skin gelatin, 0.5% Triton X-100). Whole-mounts were labelled with primary antibody overnight at 4°C, washed in PBS for 1 h at room temperature, and with secondary antibodies containing DAPI (1 µg/ml diluted 1:2000) for 2 h at room temperature (antibodies are described in Table S4). Whole-mounts were mounted on coverslips with glycerol and imaged with a Nikon A1 upright confocal using the NIS-Elements software package to stitch multiple 1024×1024 images. Since wounds were only 2 mm in diameter we could capture the entire width of each wound in one whole-mount (60 µm section) and, by analysing multiple whole-mounts from each wound, we could readily quantitate all the HFs per wound.

### Flow cytometry

Fibroblasts were isolated as previously described (Jensen et al., 2010). Briefly, back skin was isolated and incubated in dispase-trypsin solution at 37°C for 1 h. Dermis was separated from the epidermis, transferred to 0.25% collagenase in FAD medium, minced and incubated at 37°C for 1 h. The digest was passed through a 70 µm filter before centrifugation. For cell sorting, cells were washed with PBS and sorted using a BD FACSAria Fusion. For FACS analysis, fibroblasts were incubated for 1 h with antibodies (Table S4), washed with PBS and analysed with a BD FACSCanto TMII. All data analysis was carried out with FlowJo 9.8.5.

### Microarrays

Neonatal (P2) and P50 back skin PDGFRaH2BeGFP fibroblast microarrays were published previously (GEO GSE32966) (Collins et al., 2011). To generate back and tail dermis microarrays from P1 mice (C57BL6/CBA F1), tail skin was incubated in 5 mM EDTA in PBS at 37°C for 1 h, whereas back skin was incubated in dispase-trypsin solution at 37°C for 1 h. Tail and back dermis were separated from the epidermis with forceps, washed once in PBS and digested as described above. Single-cell suspensions were washed three times in PBS before cells were lysed in Trizol (Invitrogen) and RNA was purified using Qiagen RNeasy columns. RNA was extracted from triplicate mice. cDNA was amplified from purified RNA and hybridised to Affymetrix MG430.2A arrays by the Cancer Research UK Patterson Institute Affymetrix Genechip Microarray Service. Array images were produced by the Affymetrix PICR 3000 scanner and analysed as CEL files using GeneSpring 13X (Agilent Technologies). RMA normalisation was used and the bottom twentieth percentile of genes (i.e. the 20% of genes with the lowest expression levels) were excluded from subsequent analysis.

### Quantitation and statistical analysis

Statistical analysis was performed with GraphPad Prism 6 software. Unless stated otherwise, statistical significance was determined by unpaired *t*-test for biological effects with an assumed normal distribution. For unbiased cell identification with DAPI, Ki67, TOPH2BeGFP or PDGFRaH2BeGFP labelling, nuclear staining was quantified using the Spot detector plugin of Icy software (version 1.6.0.0). To quantify Lrig1^+^ and Sca1^+^ cells in the wound bed, mean fluorescence was determined with Icy software (version 1.6.0.0) and normalised to background.

To quantitate new HFs, at least eight 60 µm sections per wound covering the entire wound bed were stained with the indicated antibodies and HFs per wound bed counted. Total and dermal layer fibroblast density was quantitated in PDGFRaH2BeGFP whole-mount sections by counting EGFP^+^ cells in the dermis between adjacent HFs (30 inter-HF regions scored per biological replicate) and in the wound bed (three wound beds per biological replicate) at the indicated time points.

### PCR analysis

Flow cytometry was used to sort tdtomato^+^ and tdtomato^−^ cells. DNA was prepared from sorted cells and whole-mount sections with DNA lysis buffer (100 mM Tris-Cl pH 8.8, 50 mM NaCl, 5 mM EDTA pH 8.0, 0.2% SDS, 0.2 mg/ml proteinase K), precipitated with isopropanol, washed with 70% ethanol and resuspended in TE buffer (10 mM Tris-Cl, 1 mM EDTA, pH 8.0).

For PCR analysis, PCR Mastermix (Promega, M7502) and the primer pairs listed in Table S5 were used. PCR was performed on a C1000 Touch thermal cycler (Bio-Rad) with the following cycle conditions: 94°C for 1 min, followed by 32 cycles of 94°C for 30 s, 55°C for 1 min, 72°C for 1 min. PCR products were loaded on a 3% agarose gel and imaged with a Bio-Rad Gel Doc-XR+ system using Image Lab software (version 5.2).

### Calculation of cell division rate

We measured the body length (from between the scapulae to the tail base), width (at the widest point) and dermis depth (from back skin sections) of P2 and P50 mice (*n*=3 biological replicates) (Table S3). We used these measurements to obtain the approximate body size, represented as a cylinder (Fig. 4A). The total mouse volume can be approximated by calculating the cylinder volume *V*:
(1)
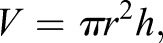
where the radius *r* represents half of the body width and *h* the animal height. The dermis volume *dv* corresponds to the difference between the total volume and the inner volume when the radius is the animal total radius minus the dermis depth *dd*:
(2)
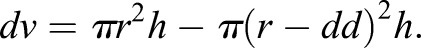
Let *N* be the number of fibroblasts in P50 occupying *dv*_P50_, *N*_0_ the number of fibroblasts in P2 occupying *dv*_P2_ and *n* the predicted number of cell divisions. If we assume exponential cellular growth then:
(3)
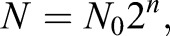

(4)
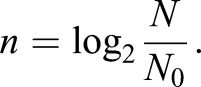


The total number of fibroblasts at a certain developmental stage can be approximated by multiplying the dermis volume *dv* by the fibroblast density *ρ*. From here we expect:
(5)
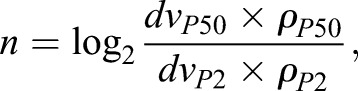


giving *n*=1.3128. Based on this result we estimate that dermal fibroblasts undergo one cell division between P2 and P50.
